# Melatonin-induced physiology and transcriptome changes in banana seedlings under salt stress conditions

**DOI:** 10.3389/fpls.2022.938262

**Published:** 2022-09-06

**Authors:** Junya Wei, Jinhao Liang, Debing Liu, Yuewei Liu, Guoyin Liu, Shouxing Wei

**Affiliations:** ^1^Tropical Crops Genetic Resources Institute, Chinese Academy of Tropical Agricultural Sciences, Haikou, China; ^2^Applied Science and Technology College, Hainan University, Haikou, China; ^3^Forestry College, Hainan University, Haikou, China

**Keywords:** melatonin, banana, physiological change, transcriptomic analysis, salinity

## Abstract

Soil salinization poses a serious threat to the ecological environment and agricultural production and is one of the most common abiotic stresses in global agricultural production. As a salt-sensitive plant, the growth, development, and production of bananas (*Musa acuminata* L.) are restricted by salt stress. Melatonin is known to improve the resistance of plants to stress. The study analyzed the effects of 100 μM melatonin on physiological and transcriptome changes in banana varieties (AAA group cv. Cavendish) under 60 mmol/l of NaCl salt stress situation. The phenotypic results showed that the application of exogenous melatonin could maintain banana plants’ health growth and alleviate the damage caused by salt stress. The physiological data show that the application of exogenous melatonin can enhance salt tolerance of banana seedlings by increasing the content of proline content and soluble protein, slowing down the degradation of chlorophyll, reducing membrane permeability and recovery of relative water content, increasing the accumulation of MDA, and enhancing antioxidant defense activity. Transcriptome sequencing showed that melatonin-induced salt tolerance of banana seedlings involved biological processes, molecular functions, and cellular components. We also found that differentially expressed genes (DEGs) are involved in a variety of metabolic pathways, including amino sugar and nucleotide sugar metabolism, phenylalanine metabolism, cyanoamino acid metabolism, starch and sucrose metabolism, and linoleic acid metabolism. These major metabolism and biosynthesis may be involved in the potential mechanism of melatonin under salt stress. Furthermore, some members of the transcription factor family, such as MYB, NAC, bHLH, and WRKY, might contribute to melatonin alleviating salt stress tolerance of the banana plant. The result laid a basis for further clarifying the salt stress resistance mechanism of bananas mediated by exogenous melatonin and provides theoretical bases to utilize melatonin to improve banana salt tolerance in the future.

## Introduction

Plants face various environmental phenomena especially biotic and abiotic stresses in the process of their life cycle and these adverse conditions limit their growth and development. Soil salinization poses a serious threat to the ecological environment and agricultural production and is one of the most common abiotic stresses in global agricultural production. Plants exposed to salt stress will reduce the yield of most crops, which has become a threat to human civilization, especially in areas with dense planting and population in the world as it is more affected by salinization. About 800 million hectares of land are affected by salinization according to the FAO (2017), resulting in the loss of 0.25–0.50 million hectares of farmland every year (Lema et al., 2019).

As a perennial monocotyledonous herbaceous group of the order Zingiberales, the banana is not only the most widely consumed fruit, but also the main food crop behind rice, wheat, and maize (Dash and Rai, 2016). Like other crops, banana plants are easily affected by various stresses by pathogens and suboptimal cultivation environments because the banana is very sensitive to salt stress as a salt-sensitive plant. Salt stress will slow down the growth of the banana plant and reduce the yield and quality. At the same time, unreasonable irrigation also causes secondary salinization of soil in the banana garden. Under the condition of salt stress, the yield of bananas decreased by about 50% (Israeli et al., 1986), and the plant height decreased by about 75% (Yano-Melo et al., 2003), which seriously restricted the sustainable development of the banana plant. Therefore, it is critical to enhance the salt tolerance of banana plants to improve their growth and production.

Melatonin (*N*-acetyl-5-methoxytryptamine) is a crucial pleiotropic molecule that was first found in plants in 1995 (Dubbels et al., 1995). It is found to play a significant function in the process of plant development, including regulating seed germination, root growth, senescence, and so on (Zhang et al., 2013; Liang et al., 2015). Furthermore, melatonin also acts as a magnificent role in different aspects of the physiological processes to help plants respond to different types of stress-containing salinity (Yu et al., 2018; Li et al., 2019a), cold (Li et al., 2016), high temperatures (Xu et al., 2010), drought (Li et al., 2019b), heavy metals (Liu et al., 2015), pathogen infection (Yin et al., 2013), and UV-B radiation (Afreen et al., 2006). As a variety of important plant growth regulators, melatonin can improve the tolerance of plants to abiotic stresses, such as drought, salinity, and alkali, whether through the external application or endogenous induction (Zuo et al., 2014). Exogenous melatonin treatment could improve the antioxidant capacity of cabbage by increasing anthocyanin content (Zhang et al., 2006). Melatonin regulates the expression of the potassium channel protein gene by regulating reactive oxygen species signal and activating calcineurin B-like 1-interacting protein kinase 23 pathway to improve the salt tolerance of apple (Li et al., 2016). Melatonin also interacts with some plant hormones and regulates gene expression related to indole-3-acetic acid (IAA), gibberellin, cytokinin, abscisic acid, and ethylene metabolism in plants (Arnao and Hernández-Ruiz, 2018). In cucumbers, melatonin regulates the expression of genes related to GA and ABA synthesis in cucumber seedlings, thereby, increasing GA content and reducing ABA content, to reduce the inhibitory effect of a high salinity environment on cucumber seedlings (Zhang H. J. et al., 2014). However, whether melatonin could enhance the salt stress of the banana plant and its molecular mechanism for salt resistance remain unclear in bananas. Transcriptome analysis with high-throughput sequencing has been widely used to study the abiotic stress tolerance of plants. Through transcriptome analysis, we can understand the response genes or key pathways that play an important role in the abiotic or biological stress of these plants (Chen et al., 2014). In this study, growth, physiological, and transcriptional analysis methods were used to explore the physiological response and possible molecular mechanism of melatonin in banana salt tolerance. The purpose of this study was to explore the enhancement of stress resistance of banana seedlings mediated by melatonin by analyzing the related physiology and transcriptome of banana plants. This study will also provide a good theoretical basis for the use of exogenous melatonin to improve plant stress resistance.

## Materials and methods

### Plant material and treatments

The banana variety (AAA group cv. Cavendish) used for the experiment is widely and presently cultivated in tropical and subtropical regions in China (Zhang X. et al., 2014). In this study, the undeveloped five-leaf banana seedlings with uniform phenotype were used as experimental materials, and 1/2 Hogland nutrient solution was used to irrigate the seedlings to maintain a constant nutrient concentration. After 1 week, three treatments were designed with banana seedlings by irrigating different solutions as follows: (1) Irrigate 1/2 Hogland nutrient solution with 60 mmol/l NaCl to the banana seedlings as salt stress treatment (S), (2) Irrigate 1/2 Hogland nutrient solution added with 60 mmol/l NaCl and 100 μM melatonin to the banana seedlings as melatonin salt treatment (MS), and (3) Irrigate 1/2 Hogland nutrient solution to the banana seedlings with 0 mmol/l NaCl treatment as control (CK). The selection of concentrations of both NaCl and melatonin was based on our previous experimental results (Liu et al., 2020). The phenotypic changes of plants were observed every day, and the height and second leaf length of plants treated with different treatments were measured on the 10th day. At the same time, the leaves of the same part were taken and divided into two parts: one part was used for related physiological analysis, and the other part was quickly frozen with liquid nitrogen and stored in a refrigerator at −80°C for subsequent cDNA library preparation and RNA sequence analysis. There were three biological replicates in each treatment.

### Measurements of physiological characterization

The plant height and the second leaf length of the three treatments were measured using a ruler. For the relative water content of the leaves, first, select 0.250 g banana seedling leaves, put the leaves in deionized water for 24 h, weigh the weight of the soaked leaves as W_*T*_, and put them in a 105°C oven for 15 min, and then put them in a 70°C incubator for drying until the constant weight is W_*D*_; calculated according to the following formula: relative water content (%) = (0.25 − W_*D*_)/(WT − W_*D*_) × 100 (Li, 2000). For chlorophyll content determination, cleaned the leaves with distilled water, wiped them with filter paper, cut off the main vein, accurately weighed 0.2 g, put it into a 25-ml volumetric flask, added 20 ml of 95% ethanol solution, soaked it in the dark for 12 h, and then fixed the volume to 25 ml with 95% ethanol solution after 12 h. The chlorophyll content was calculated by the following formula: Total chlorophyll content = (20.21 D_645_ + 8.02 D_663_) × V/(1,000 × m) (Zhang and Zhai, 2003). D_663_ and D_645_ are the absorbance values at 663 nm and 645 nm, respectively; V is the volume of the solution to be measured (ml); m is the fresh mass of leaves (g). The permeability of leaves plasma membrane, the soluble sugar content, and proline content were measured by conductometer, anthrone colorimetry, and the Acid-ninhydrin method, respectively (Gao, 2006). Selected and weighed 0.5 g leaves of banana seedlings, ground with 8 mL of 50 mmol/l phosphate buffer (PBS) solution with pH 7.8, washed it into a 50-ml centrifuge tube, and then centrifuged at 4°C 10,000 r/min for 20 min. The supernatant was used to determine soluble protein content, malondialdehyde content, superoxide dismutase content, and peroxidase content (Gao, 2006). Soluble protein content, malondialdehyde (MDA) content, SOD, and POD activity were measured as described (Zhang and Zhai, 2003). Each analysis has three replicates, one of which includes three seedlings.

### Illumina sequencing and data analysis

We extracted the total RNA of all 9 samples (three CK samples, three S samples, and three MS samples) from banana leaf samples using the RNeasy plant kit (Qiagen, Dusseldorf, Germany). The concentration and purity of RNA samples were measured by NanoDrop 2000 (Thermo Fisher Scientific, Wilmington, DE, United States). The integrity of RNA was evaluated by the RNA Nano 6000 Assay Kit of the Agilent Biological analyzer 2100 system (Agilent Technology, Santa Clara, CA, United States). The total RNA amount of each sample was 1 μg and used as input material. According to the manufacturer’s recommendations, the NEBNext UltraTM RNA library for Illumina (NEB, Waltham, MA, United States) was used to prepare the kit to generate the sequencing library, and the index code was added to the attribute sequence of each sample. Cluster index coded samples on the cBot cluster Generation System using Truseq PE Cluster kit v4-cBot-HS (Illumina). After the cluster is generated, the library preparation was sequenced and paired-end reads are generated. We extracted the total RNA of all nine samples (three CK samples, three S samples, and three MS samples) from banana leaf samples using the RNeasy plant kit (Qiagen, Dusseldorf, Germany). The concentration and purity of RNA samples were measured by Nanodrop 2000. The integrity of RNA was evaluated by the RNA nano 6000 test kit by Agilent biological analyzer 2100 system (Agilent Technology, Santa Clara, CA, United States). The total RNA content of each sample was 1 μg and used as input material. Use NEBNext UltraTM RNA Library (NEB in the United States) to generate the sequencing library, and the index code was added to the attribute sequence of each sample. Cluster index coded samples on the cBot cluster Generation System using Truseq PE Cluster kit v4-cBot-HS (Illumina). After the cluster is generated, the library preparations are sorted and paired-end reads are generated. The nine gene expression libraries were named C-1, C-2, C-3, S-1, S-2, S-3, MS-1, MS-2, and MS-3. RNA sequence libraries were generated using Illumina Hiseq™ 2000 Sequencing by a biomarker biotechnology company (Beijing, China). The raw data in fastq format was first handled through an internal Perl script. Clean data could be obtained by deleting reads with adapters, reads with poly-N, and low-quality reads from the original data. At the same time, Q20, Q30, GC content, and sequence repeat level were calculated in clean data. Deleted adapter sequence and low-quality sequence reads from the dataset and the original sequence were converted to a clean read. These clean readings were then mapped to banana reference genome sequences and gene functions analysis was annotated through the NR, Nt, Pfam, GO, and other databases.

### Identification of differentially expressed genes and functional enrichment analysis

We performed differential expression analysis using DESeq2 software (Anders and Huber, 2010). The threshold of significant differential expression was set as the FDR < 0.01 and Fold Change ≥ 2. Gene Ontology (GO) database and homologous group clustering (COG) database, while the Kyoto Encyclopedia of Genes and Genomes (KEGG) database was used to search for differentially expressed genes (DEGs). Then, the differentially expressed genes (DEGs) were enriched by Gene Ontology (GO) analysis based on Wallenius non-central hypergeometric distribution (Young et al., 2010). In addition, the statistical enrichment of differentially expressed genes in the KEGG pathway was tested by KOBAS (Mao et al., 2005).

### Real-time quantitative PCR analysis

A random selection of ten significant difference DEGs was chosen to confirm the RNA-seq results by RT-qPCR using the gene-specific primers listed in Supplementary Table 1. The UBQ gene was used as the internal reference gene for the results of the qRT-PCR analysis. The relative expression levels were determined by 2^–ΔΔCt^ methods (Schmittgen and Livak, 2008). The data were subjected to ANOVA with Duncan’s multiple range test at *P* < 0.05. Each reaction was performed in triplicates.

## Results

### Growth and phenotypes of banana seedlings under salt stress by melatonin

**FIGURE 1:**
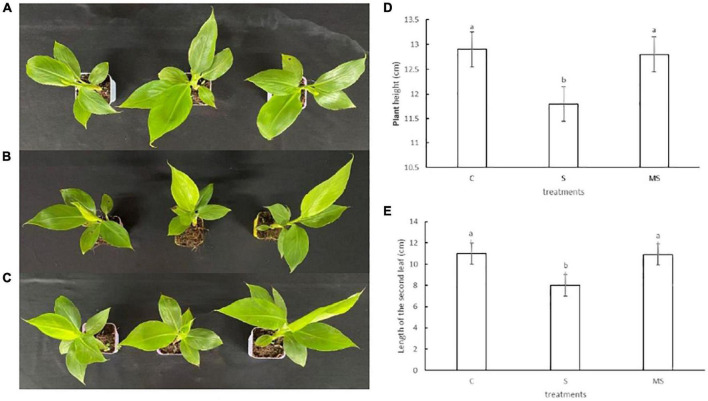
Phenotypes traits of banana seedlings on the 10th day under different treatments. Experiments were repeated three times and bars with different letters are significantly different according to Duncan’s multiple range tests (*P* < 0.05). **(A)** Images of control phenotypes. **(B)** Images of 60 mM NaCl phenotypes. **(C)** Images of 60 mM NaCl plus 100 μM melatonin phenotypes. **(D)** Plant height. **(E)** The second true leaf length.

To accurately evaluate the response of banana seedlings under 0 mmol/l NaCl, 60 mM NaCl, and 60 mM NaCl plus 100 μM melatonin treatments, young banana seedlings at the five-leaf state with constant growth were treated with three different treatments. Compared with the 0 mmol/l NaCl treatment, the banana seedlings treated with 60-mM NaCl grew slowly. With the extension of salt stress time, the leaves turned brown, appeared the symptoms of yellowing and leaf edge necrosis, and gradually withered. However, the banana seedlings treated with 60 mM NaCl plus 100 μM melatonin showed larger or higher leaves than those treated with 60 mM NaCl, which had a similar phenotype to the 0 mmol/l NaCl treatment. Thus, the growth results imply that 60 mM NaCl salt stress inhibited the growth of banana seedlings, while 100 μM of melatonin could alleviate the symptoms of 60 mM NaCl salt stress of banana seedlings (Figure 1).

### Physiological properties of banana seedlings under salt stress by melatonin

To accurately assess the physiological properties of exogenous melatonin on banana seedlings under salt stress, the soluble protein content, proline content, chlorophyll content, relative water content, membrane permeability, MDA, SOD, and POD of banana seedlings were monitored. The results showed that there were obvious differences in the physiological characteristics of banana seedlings under different treatments.

**FIGURE 2:**
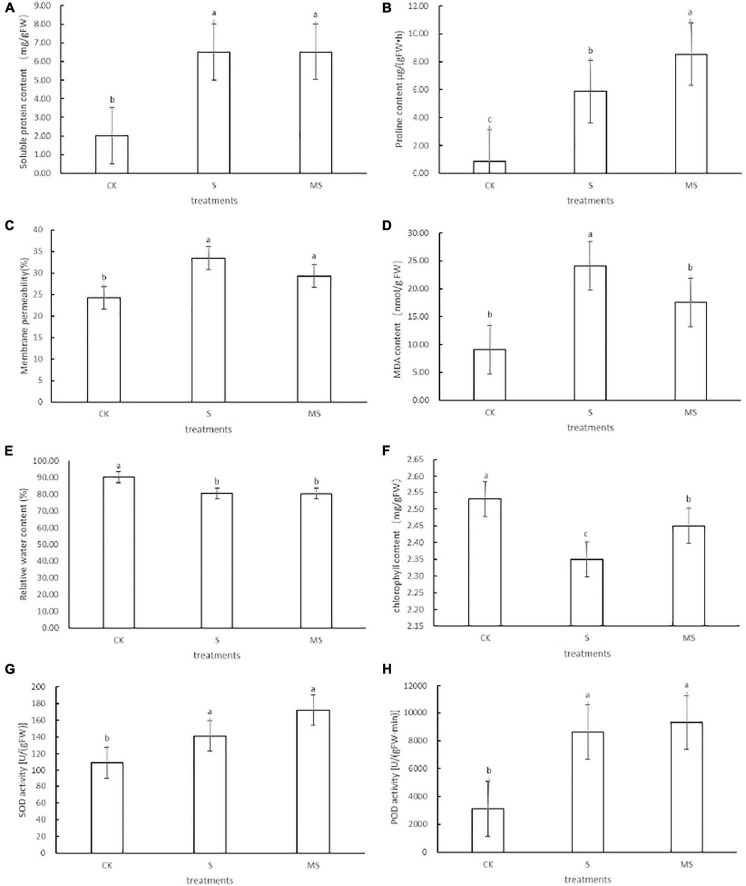
Effects of different physical traits of DB banana seedlings exposed to different treatments on the 10th day. **(A)** Soluble protein content. **(B)** Proline content. **(C)** Membrane permeability. **(D)** MDA content. **(E)** Relative water content. **(F)** Chlorophyll content. **(G)** SOD content. **(H)** POD content. CK, treatment with half-strength Hoagland growth nutrient solution added with 0 mmol/L NaCl; S, treatment with half-strength Hoagland growth nutrient solution added with 60 mmol/L NaCl; MS, treatment with half-strength Hoagland growth nutrient solution added with 60 mmol/L NaCl plus 100 μM melatonin. The different letters represent significant differences at *p* < 0.05.

Salt stress can cause osmotic stress in plants. To adapt to salt stress and prevent water loss, plants often built-up different osmotic regulator substances (such as soluble protein) to raise the concentrations of cellular fluids (Yang and Guo, 2018). Compared with the control group, banana seedlings exposed to 60 mM of NaCl could increase the soluble protein content. Compared with banana seedlings treated with 60 mM of NaCl, these parameters were further increased when salt-stressed seedlings were treated with 60 mM of NaCl plus 100 μM melatonin (Figure 2A). To explore the mechanism of melatonin participating in proline biosynthesis, we analyzed the changes in proline content in banana seedlings treated with 60 mM of NaCl plus 100 μM melatonin and with 60 mM of NaCl only. Compared with the 0 mM NaCl treatment group, banana seedlings exposed to 60 mM of NaCl treatment could increase proline content. Compared with banana seedlings treated with 60 mM of NaCl treatment, these parameters were further increased when salt-stressed seedlings were treated with 60 mM of NaCl plus 100 μM of melatonin (Figure 2B). These results showed that under salt stress, using 100 μM of melatonin could significantly increase the accumulation of soluble protein and proline content in banana seedlings, effectively regulating intracellular water balance, protecting cell membrane structure, and reducing cell damage. We also evaluated the effects of melatonin on membrane permeability and membrane lipid peroxidation of banana seedlings under salt stress by measuring membrane permeability (%) and MDA concentration. Compared with the 0 mM NaCl treatment, the membrane permeability and MDA content of banana seedlings exposed to 60 mM of NaCl increased. Banana seedlings treated with 60 mM of NaCl and 100 μM of melatonin showed less increase in membrane permeability and MDA content than plants treated with only 60 mM of NaCl (Figures 2C,D). The results showed that melatonin could significantly inhibit the accumulation of MDA content in banana seedling cells and alleviate the damage to cell membranes caused by the salt-stress membranes. Under salt stress, compared with the 0 mM of NaCl treatment, the relative water content and chlorophyll content decreased by 10 and 7.1%, respectively, while after the application of exogenous melatonin, the relative water content, and chlorophyll content recovered by 10 and 54.2%, respectively (Figures 2E,F). Chlorophyll biosynthesis is an important indicator to determine the salt tolerance of plants. To explore the role of melatonin in photosynthetic pigment biosynthesis under 60 mM of NaCl and control, we estimated the concentration of chlorophyll (Figure 2E). Compared with 60 mM of NaCl plus 100 μM melatonin treatment and the 0 mM of NaCl treatment, the chlorophyll values of banana seedlings treated with 60 mM of NaCl treatment were the lowest. It showed that melatonin could alleviate the degradation of chlorophyll, which was consistent with Siddiqui’s experimental results (Siddiqui et al., 2012). Under adverse conditions, plants will use their enzyme antioxidant systems (such as SOD and POD) to remove excess ROS to protect plant cells from oxidative damage (Munns and Tester, 2008). To further evaluate the mechanism of melatonin-induced salt tolerance in banana seedlings, we also examined the levels of superoxide dismutase (SOD) and antioxidant enzyme (POD) in banana seedlings under the three treatments. It can be seen from the results shown that banana seedlings treated with 60 mM of NaCl showed increased activities of SOD and POD compared with their respective controls (Figures 2G,H). Compared with the 60 mM of NaCl treatment, the application of melatonin to salt-stressed seedlings (60 mM NaCl plus 100 μM melatonin) further enhanced the activities of these enzymes. POD functions as an antioxidant enzyme that plays a significant role in hydrogen peroxide detoxification and lignin biosynthesis (Chibbar and Vanhuystee, 1984; Gao et al., 2010). Our experience data indicated that exogenous melatonin could further increase SOD and POD levels in banana seedlings under salt stress. The above results showed that the application of exogenous melatonin could induce salt stress by increasing the content of proline content and soluble protein, slowing down the degradation of chlorophyll, improving membrane permeability, decreasing the relative water content, reducing the accumulation of MDA, and enhancing antioxidant defense activity of banana seedlings.

### RNA sequencing and data analysis

To have a comprehensive survey of the molecular mechanism of melatonin on banana seedlings under salt stress conditions, we constructed 9 cDNA libraries, which were three replicates of the leaves of 0 mmol/l NaCl (CK), 60 mM of NaCl (S), and 60 mM of NaCl plus 100 μM melatonin (MS), using the Illumina sequencing platform and generated nine sub transcriptomes. The map of Spearman’s Correlation Coefficient between different sample pairs states clearly that each sample was reliable and had good reproducibility (Supplementary Table 2). The statistical data of banana transcriptome are shown in Supplementary Table 3. A total of 498,004,468 readings were generated from 9 cDNA libraries. By removing the low-quality readings of banana seedlings, 90685718 (CK), 89615843 (S), and 68313446 (MS), clean readings were obtained, with Q20 > 98.1% and Q30 > 94.7% (Supplementary Table 3). The results showed that the transcriptome sequencing data could be used for further analysis.

### Identification of differentially expressed genes among different treatments

**FIGURE 3:**
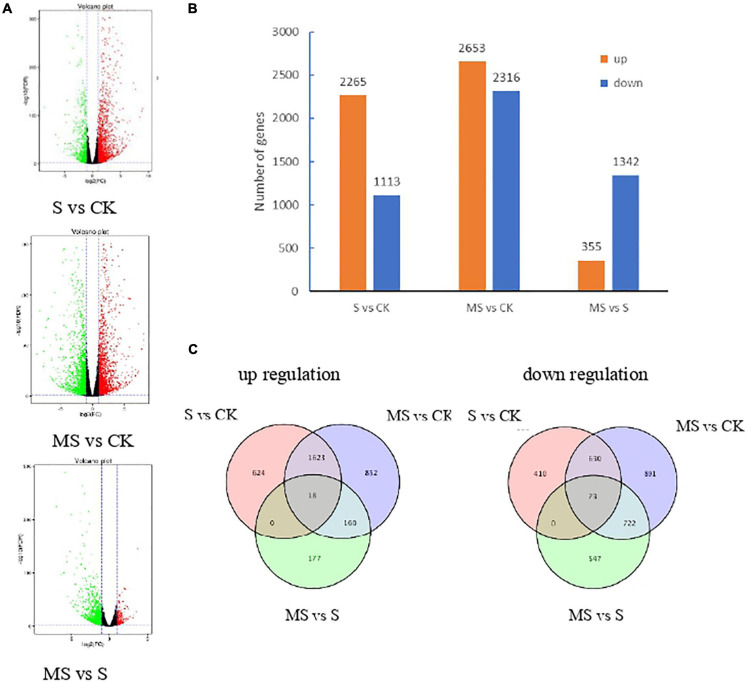
Transcriptional variations in BD banana plants under different treatments. **(A)** Volcano plots analysis of the DEGs in different treatments. **(B)** The number of up- and down-regulated genes in different treatments. **(C)** Venn diagrams showed the proportions of the up- and down-regulated genes in three treatments.

We carried out differential expression analysis using the DESeq2 (Anders and Huber, 2010). The false discovery rate (FDR) was used to determine the threshold of the *P*-value, which was used to calculate the significance of the differences. Differential expression genes (DEGs) were identified between each pair of combinations treated with CK and MS by the thresholds of FDR (*q* value) < 0.01 | log2 (fold change)| ≥ 2. To observe the number of transcripts was significantly regulated in different treatments, the volcano plots analysis of the DEGs was constructed in Figure 3A. The red dot represents the significantly upregulated EDGs, while the green dot represents the significantly downregulated EDGs. The results showed that compared with salt treatment, the transcription of DEGs in melatonin treatment was less, which was consistent with the results of rape seedlings under salt stress (Tan et al., 2019).

A total of 3,378 DEGs were identified in S compared with CK. A total of 4,969 DEGs were identified in MS vs. CK. A total of 1,697 DEGs were highly expressed in MS compared to S (Supplementary Table 4; Figure 3B). Respectively 2,265, 2,653, and 355 upregulated DEGs and 1,113, 2,316, and 1,342 downregulated DEGs in the comparison of S vs. CK, MS vs. CK, and MS vs. S. From the data we can know that there were more upregulated DEGs in the comparisons of S vs. CK, MS vs. CK than in the comparisons of MS vs. S. We also compared the proportions of the up-regulation and down-regulation DEGs among S vs. CK, MS vs. CK, and MS vs. S by constructing the Venn diagrams (Figure 3C). There were 18 upregulated DEGs and 73 downregulated among the three comparative groups. It is worth noting that there were more differentially expressed genes in MS vs. CK than in S vs. CK, indicating that melatonin may induce more stress-related genes in a salt-stress situation.

### Gene ontology function analysis of differentially expressed genes among different treatments

**FIGURE 4:**
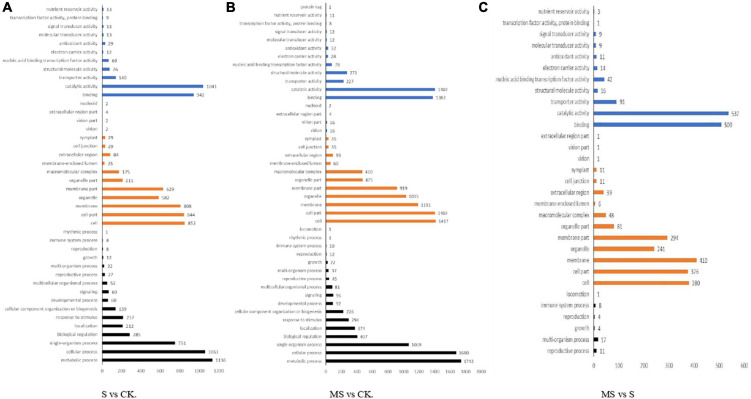
Gene ontology (GO) classification analysis of the DEGs in different comparison group. **(A)** S vs. CK. **(B)** MS vs. CK. **(C)** MS vs. S.

We classified these DEGs into the following three categories according to GO categories: biological processes, cellular components, and molecular functions to clarify their functions of them. We also classified the functions of transcripts in different treatments by GO analysis with a threshold of *p* < 0.01. Between S and CK comparison, 3,378 DEGs were divided into three primary categories (biological processes, cellular components, and molecular functions) and were further separated into 42 subgroups. There were 37.96% DEGs classified as “biological process category,” 40% of DEGs classified as “cellular component category,” and 22.04% of DEGs were classified as “molecular function category” among these DEGs. Among them, biological processes category mainly included “metabolic process,” “cellular process,” “monomer process,” “biological regulation,” and “localization.” Cell components category mainly included “cell,” “cell part,” “membrane,” “organelle,” and “membrane part.” Molecular functions category mainly included “binding,” “catalytic activity,” “transporter activity,” “structural molecular activity,” and “nucleic acid binding transcription factor activity” (Figure 4A). Between MS and CK comparison, 4,969 DEGs were divided into three primary categories (biological processes, cellular components, and molecular functions) and were further separated into 44 subgroups. There were 36.76% DEGs classified as “biological process category,” 42.63% of DEGs were classified as “cellular component category,” and 20.61% of DEGs were classified as “molecular function category” among these DEGs. The main GO classifications were the same as S vs. CK comparison (Figure 4B). Between MS vs. S comparison, 1,697 DEGs were divided into three primary categories (biological processes, cellular components, and molecular functions) and were further separated into 31 subgroups. Approximately 1.41% of DEGs were classified as “biological process category,” 59.62% of DEGs were classified as “cellular component category,” and 26.28% of DEGs were classified as “molecular function category” among these DEGs. Interestingly, the main biological processes category mainly included “reproductive process,” “multi-organism process,” “growth,” “reproduction,” and “immune system process” that were different from the other two treatments (Figure 4C).

### Gene ontology enrichment analyses of differentially expressed genes among different treatments

We had known the functions of these DEGs among different treatments through the GO database and gained the enrichment analysis of DEGs in banana seedlings. In the comparison between S and CK, the top three terms of GO enrichment in the biological process were “photosynthesis, light capture in photosystem I (go:0009768),” “protein chromophore linkage (go:0018298),” and “phosphorus ion transport (go:0006817).” The top three terms of GO enrichment in molecular function were “chlorophyll-binding (go:0016168),”\“*O*-methyltransferase activity (go:0008171),” and “motility activity (go:0003774)”; and the top three go enrichment terms in cell components were “photosystem I (go:0009522),” “myosin complex (go:0016459),” and “photosystem I reaction center (go:0009538).” In the comparison between MS and CK, the top three GO enrichment terms in the biological process were “translation (GO:0006412),” “photosynthesis, light harvesting in photosystem I (GO:0009768),” and “protein-chromophore linkage (GO:0018298)”; the top three GO enrichment terms in the molecular function were “structural constituent of ribosome (GO:0003735),” “chlorophyll-binding (GO:0016168),” and “rRNA binding (GO:0019843)”; the top three GO enrichment terms in the cellular component were “ribosome (GO:0031408),” “cytosolic large ribosomal subunit (GO:0006817),” and “photosystem I (GO:0010466).” In the comparison between MS vs. S, the top three GO enrichment terms in the biological process were “oxylipin biosynthetic process (GO:0008171),” “phosphate ion transport (GO:0097027),” and “negative regulation of peptidase activity (GO:0010997),” the top three GO enrichment terms in the molecular function were “*O*-methyltransferase activity (GO:0005887),” “anaphase-promoting complex binding (GO:0016459),” and “motor activity (GO:0009505)”; the top three GO enrichment terms in the cellular component were “integral component of plasma membrane,” “myosin complex,” and “plant-type cell wall.”

### Kyoto encyclopedia of genes and genomes pathway analysis of differentially expressed genes among different treatments

To comprehensively understand the lively biological pathways in DEGs among different treatments and determine the main pathways of melatonin related, the KEGG pathway enrichment analysis was performed according to expression profile. Supplementary Table 4 showed the main differential biological pathways of these DEG in banana seedlings. These results clearly stated that 1,076 DEGs were annotated to 112 metabolic pathways in the comparison between S and CK. Among them, 637 upregulated DEGs were annotated with 92 metabolic pathways, and 439 downregulated DEGs were annotated with 90 metabolic pathways. Among the 112 metabolic pathways, 22 metabolic pathways were annotated as upregulated DEGs, 20 metabolic pathways were annotated as downregulated DEGs, and 69 metabolic pathways were annotated as both upregulated and downregulated genes. There were 1,779 DEGs annotated to 118 metabolic pathways in the comparison between MS and CK. Among them, 712 upregulated DEGs were annotated with 105 metabolic pathways, and 1,067 downregulated DEGs were annotated with 103 metabolic pathways. Among the 118 metabolic pathways, 15 metabolic pathways were annotated as upregulated DEGs, 13 metabolic pathways were annotated as downregulated DEGs, and 90 metabolic pathways were annotated as both upregulated and downregulated genes. There were 629 DEGs annotated to 104 metabolic pathways in the comparison between MS vs. S. Among them, 80 upregulated DEGs were annotated with 47 metabolic pathways, and 549 downregulated DEGs were annotated with 97 metabolic pathways. Among the 114 metabolic pathways, 9 metabolic pathways were annotated as upregulated DEGs, 57 metabolic pathways were annotated as downregulated DEGs, and 39 metabolic pathways were annotated as both upregulated and downregulated genes.

Through pathway enrichment analysis (*p* < 0.01), we identified the top ten significant enrichment pathways between the three different treatments (Supplementary Table 5). There were many significant pathways for plants against the abiotic and biological stresses reported in previous studies, such as photosynthesis and photosynthesis antenna protein (ko00196), phenylpropanoid biosynthesis (ko00940), ribosome (ko03010), starch and sucrose metabolism (ko00500), an amino sugar, nucleotide sugar metabolism (ko00520), and so on (Goyal et al., 2016; Zhang et al., 2017), could be found in Supplementary Table 5. The phenylpropane pathway could play a function in plant growth and stress response by synthesizing different plant secondary metabolites. Plants also accumulated large amounts of flavonoids in adverse environments (Winkel-Shirley, 2002). Furthermore, two pathways, Phenylpropanoid biosynthesis (ko00940) and Flavonoid biosynthesis (ko00941) were simultaneously, significantly enriched in all three treatments. The phenylpropane biosynthesis pathway is the main component of plant special metabolism, which can provide anthocyanins for pigmentation and flavonoids to prevent UV light damage, etc. (Ferrer et al., 2008).

To further identify melatonin-mediated salt stress response genes involved in certain specific pathways, the important pathways identified through KEGG enrichment analysis were annotated as upregulated DEGs in the comparison between MS and S. Glycosphingolipid biosynthesis, plant hormone signal transduction, and flavonoid biosynthesis were significantly enriched (Supplementary Table 6). We found that the key enzyme “9-cis-epoxy carotenoid dioxygenase”(Ma05_g02500/Ma05_g15540, EC:1.13.11.51) in the “carotenoid biosynthesis pathway” was upregulated, which was reacted with a substrate for the synthesis of abscisic acid (ABA) in the carotenoid biosynthesis pathway (Lu and Li, 2008). Cytokinin dehydrogenase, the key enzyme in Zeatin biosynthesis was also found upregulated (Ashikari et al., 2005). The analysis of these KEGG pathways will help to study the role of melatonin in the salt stress response of banana seedlings.

### Genes involved in plant hormone signal transduction

**FIGURE 5:**
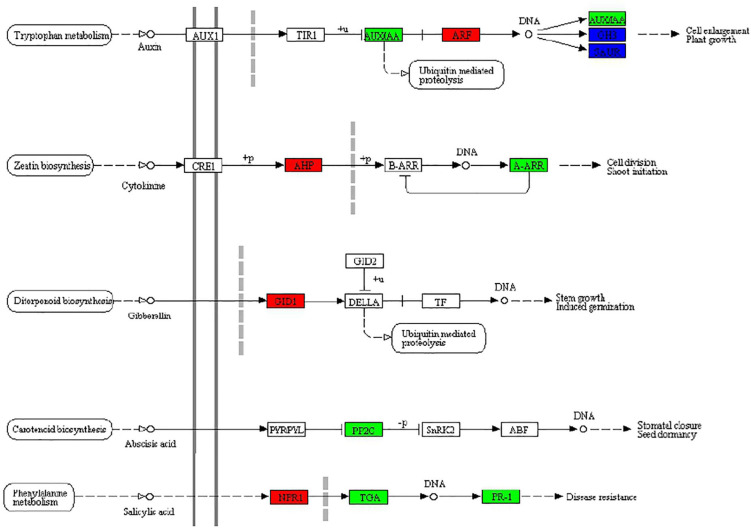
Effects of exogenous melatonin on plant hormone signal transduction (ko04075) in BD banana seedlings under salt stress. Response in plant hormone signal transduction to exogenous melatonin treatment, upregulated genes marked in red box, downregulated genes marked in green box, and both upregulated and downregulated marked in blue box, white represents no significant change.

According to the above KEGG enrichment analysis results, we make further efforts to analyze the DEGs related to the phytohormone signal transduction pathway (ko04075) in MS vs. S comparison including auxin, cytokinin, and abscisic acid in BD banana (Figure 5). Auxin, as a small organic acid, plays an important function in plants, affecting cell division, cell elongation, and cell differentiation, and has a significant impact on the final shape and function of all higher plant cells and tissues (Ljung, 2013). In our study, the ARF (AUXIN RESPONSE FACTOR) functions as a transcriptional activator of auxin-regulated genes (Ma06_g09030; Ma06_g30810) and was upregulated between MS and S (Mano and Nemoto, 2012). Cytokinins participated in the growth and development of almost all kinds of plants. Cytokinin signaling mechanism consists of the following part: AHKs (Histidine kinases), AHPs (histidine-containing phosphotransfer protein), and ARRs (response regulators) in the higher plant (Suzuki et al., 2000; Veerabagu et al., 2012). Among which, A-ARR (type-A response regulators) act as negative regulators and B-ARR (type B response regulators) act as positive regulators which regulated downstream activity in the cytokinin signaling pathway. Between MS vs. S, the expression of two genes in banana seedlings homologous to A-ARR (Ma06_g19860 Ma08_g29720) was downregulated and homologous to AHP (Ma10_g14170, Ma11_g10050) was upregulated, which may be conducive to the growth of banana seedlings. Gibberellin (GA) is a major regulator of plant growth and development. Gibberellin (GA) receptor GIBBERELLIN-INSENSITIVE DWARF1 (GID1) inhibits the repression activity of DELLAs on GA signaling (Griffiths et al., 2006). It was revealed that the expression of gibberellin receptor GID1 (Ma06_g17630) was upregulated between MS and S, which undoubtedly promoted the growth of the banana seedling. Abscisic acid (ABA) has an essential role to regulate various physiological processes of plants especially stress physiology (Park et al., 2009). Melatonin could regulate the expression of genes related to GA and ABA synthesis in cucumber seedlings, thereby, increasing GA content and reducing ABA content, thereby reducing the inhibitory effect of a high salt environment on cucumber seedlings (Zhang et al., 2013). The downregulation of PP2C (Ma05_g31380) expression between MS and S in banana seedlings alleviated the inhibition of ABA on seedling growth, which was the same as this result. Jasmonic acid has been involved in the plant salinity response (Zhao et al., 2014). In our study, α-linolenic acid metabolism (Ko00592) and plant hormone signal transduction (Ko04075) were significantly enriched between MS and S comparison, which is the same as that of Kiwifruit (Tan et al., 2019). Salicylic acid (SA) played a significant regulatory function in a variety of physiological processes, including plant immune response (An and Mou, 2011). Salicylic acid and NPR1 could induce trans-activated TGA factors to be recruited into the defense gene promoter in Arabidopsis plants (Johnson et al., 2003). The expression of NPR1 (Ma11_g18680) was upregulated, TGA (Ma09_g18630) and PR-1 (Ma02_g15060, Ma02_g15080, Ma04_g29630, Ma04_g29640) were downregulated between MS vs. S, which may be beneficial to the ability of banana seedlings to induce salt stress.

### Transcription factors analysis of differentially expressed genes among different treatments

**FIGURE 6:**
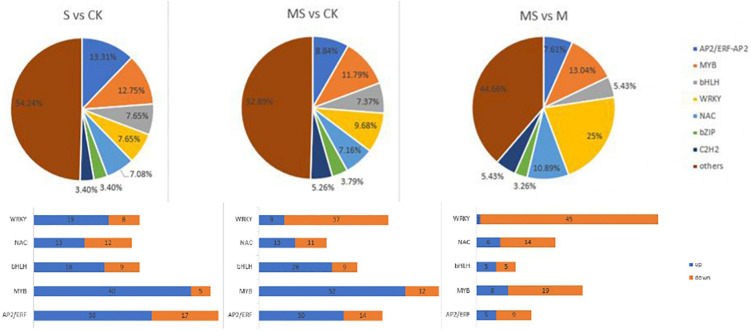
Distribution of transcription factor gene families expressed in different comparison group. S vs. CK. MS vs. CK. MS vs. S.

Transcription factors (TFs) participated in the response induced by plant stress tolerance and act as upstream regulators to regulate gene expression in the process of plant metabolic pathways (Shan et al., 2013). This study compared the expression profiles of TFs between different processes to evaluate the complex signal pathway network (Supplementary Table 7 and Figure 6). The number of genes encoding putative TFs is 353 between S and CK and these TFs were further separated into 20 groups in accordance with homolog classification. In addition, five TF families account for 50.42% of these groups, among which AP2/ERF (48 numbers), MYB (51 numbers), WRKY (27 numbers), bHLH (27 numbers), and NAC (25 numbers), played an important function in coping with salt stress tolerance. The number of genes encoding putative TFs between MS and CK was 475. According to the classification of homologs, these TFs were divided into 20 groups. In addition, the top five TF families accounted for 44.84% of these groups. MYB (64 numbers), WRKY (46 numbers), bHLH (35 numbers), AP2/ERF (42 numbers), and NAC (34 numbers), all played significant functions in melatonin and salt stress tolerance. The number of genes encoding putative TFs is 184 between MS and S of banana seedlings. According to the classification of homologs, these TFs were divided into 20 groups. In addition, the top five TF families accounted for 64.67% of these groups with WRKY (46 numbers), MYB (27 numbers), NAC (20 numbers), AP2/ERF (16 numbers), and bHLH (10 numbers). It was reported that some TFs (such as AP2/ERF, MYB, WRKY, bHLH, and NAC) had been proved to participate in plant defense response and play a major function in coping with stress responses (Alves et al., 2014; Nakashima et al., 2014). The MYB transcription factor family is found to be composed of many functionally diverse proteins found in all eukaryotes. Most MYB proteins were involved in the regulation of different cellular processes in plants and play an important role as transcription factors in various biological and abiotic stress responses (Stracke et al., 2001). The analysis results showed that 45 MYB TFs were induced between S and CK, of which 40 were upregulated and 5 were downregulated. The analysis results also showed that 64 MYB TFs were induced, including 52 upregulated and 12 downregulated between MS and CK. The analysis results also showed 27 MYB TFs that were induced including 8 upregulated and 19 downregulated between MS vs. S. There had 47 AP2/ERF TFs that were induced including 30 upregulated and 17 downregulated between S and CK in accordance with the results obtained. There had 44 AP2/ERF transcription factors induced including 30 upregulated and 14 downregulated between MS and CK. There had 16 AP2/ERF transcription factors induced, including 5 upregulated and 9 downregulated between MS vs. S. WRKY transcription factors could participate in plant’s transcriptional reprogramming to cope with different stress environments (Rushton et al., 2010). According to the results obtained, 27 WRKY TFs were induced between S vs. CK, of which 19 were upregulated and 8 were downregulated. A total of 46 WRKY TFs were induced between MS vs. CK, of which 9 were upregulated and 37 were downregulated. A total of 46 WRKY TFs were induced between MS vs. S, of which 1 was upregulated and 45 were downregulated. Previous studies have suggested that overexpression of *MusaWRKY71* in banana plants can improve the tolerance of plants to either oxidative stress or salt stress (Shekhawat and Ganapathi, 2013). We also found that there were 27 bHLH TFs induced between S and CK, of which 18 were upregulated and 9 downregulated. There were 35 bHLH TFs induced between MS and CK, of which 26 were upregulated and 9 downregulated. There were 10 bHLH TFs induced between MS and S, of which 5 were upregulated and 5 were downregulated. As plant-specific TFs, NAC TFs contain a highly conserved N-terminal domain named NAC domain. It was reported that some NAC genes were considered to be involved in salt stress response (Mahmood et al., 2016). In this study, 25 NAC TFs were induced between S (60 mM of NaCl treatment) and CK (0 mmol/l NaCl treatment), of which 13 were upregulated and 2 were downregulated. A total of 34 NAC TFs were induced between MS and CK, of which 13 were upregulated and 11 were downregulated. A total of 20 NAC TFs were induced between MS and S, of which 6 were upregulated and 14 were downregulated.

### Validation of RNA-seq data by quantitative real-time PCR analysis

**FIGURE 7:**
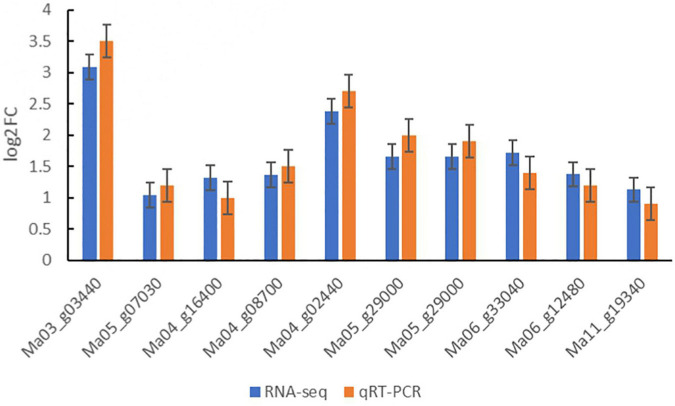
Relative expression levels of 10 genes examined by qRT-PCR. The relative gene expression levels were determined by 2^–ΔΔCT^. Transcript levels were normalized to the expression level of UBC. Data are means ± SD of *n* = 3 independent experiments.

Ten DEGs were randomly chosen from DEGs for quantitative real-time PCR analysis to further verify the reliability of sequencing data of Illumina RNA-Seq results in three treatments of banana seedlings (Figure 7; Supplementary Table 1). The results showed that the expression patterns of DEGs obtained by quantitative real-time PCR were similar to that of the RNA-seq. These results suggested that the data obtained from RNA-Seq analysis were reliable.

## Discussion

Banana is a salt-sensitive plant and soil salinity is the main environmental stress that affects the growth and productivity of bananas, which will slow down the growth of banana plants and reduce the yield and quality. As a variety of important plant growth regulators, melatonin can improve the tolerance of plants to drought, saline-alkali, or other abiotic stresses, whether by external application or endogenous induction (Zuo et al., 2014). Melatonin is known to improve the resistance of plants to adversity. Several studies have reported that melatonin could strengthen abiotic stress resistance in cucumber (Zhang H. J. et al., 2014), soybean plants (Wei et al., 2015), rice (Liang et al., 2015), and maize (Li et al., 2019c), etc. However, it is not clear whether melatonin can enhance the salt stress and salt resistance of banana seedlings. Therefore, this article studied the molecular and physiological mechanisms of melatonin on banana seedlings under salt stress, which will help to promote the growth and output of banana plants under salt stress.

To study the physiological characteristics of exogenous melatonin on banana seedlings, we accurately evaluated the soluble protein content, proline content, chlorophyll content, relative water content, membrane permeability, MDA, SOD, and POD content of banana seedlings treated with melatonin under salt stress. There are obvious differences in the physiological characteristics of banana seedlings under different treatments. The seedlings treated with 60 mM of NaCl plus 100 μM melatonin showed larger or higher leaves than seedlings treated with 60 mM of NaCl, which had a similar phenotype to the control treatment. Thus, melatonin can alleviate the symptoms of salt stress in banana seedlings (Figure 1). When plants are subjected to salt stress, they usually construct different osmoregulation substances (such as soluble protein and proline) to resist stress by increasing the concentration of cell fluid (Yang and Guo, 2018). Therefore, the contents of soluble protein and proline of banana seedlings treated with 60 mM of NaCl increased. However, the soluble protein and proline contents of banana seedlings treated with 60 mM of NaCl and 100 μM of melatonin were further increased than the control (Figures 2A,B). It is reported that a melatonin-mediated increase of proline may improve the salt tolerance of tomato seedlings (Siddiqui et al., 2019), which is consistent with the results of this study. In the meantime, we thought that exogenous melatonin could significantly increase the accumulation of soluble protein and proline in banana seedlings, effectively regulate intracellular water balance, protect cell membrane structure, and reduce cell damage under salt stress. In this study, 60 mM NaCl treatment significantly increased the membrane permeability and MDA content of banana seedlings. Melatonin treatment with 60 mM of NaCl and 100 μM of melatonin significantly reduced membrane permeability and increased MDA content (Figures 2C,D). This is consistent with the research results on naked oats under salt stress (Gao et al., 2019). The results showed that melatonin could significantly inhibit the accumulation of MDA content in banana seedling cells and alleviate the damage to the cell membrane caused by salt stress. Photosynthesis determines the growth and development of plants and the production of dry matter. Like other physiological, biochemical, and molecular processes, photosynthesis is affected by different environmental stresses (Ashraf and Harris, 2013). The results of this study showed that exogenous melatonin could alleviate the degradation of chlorophyll under salt stress (Figure 2F). This result is consistent with the study on tomatoes, which shows that exogenous melatonin maintains the stability of chlorophyll under a salt-stress situation (Siddiqui et al., 2019). The above results show that exogenous melatonin could increase proline content and soluble protein content, slow down chlorophyll degradation, reduce membrane permeability, recover relative water content, increase MDA accumulation, and improve antioxidant defense activity of banana seedlings to induce salt stress situation.

To have a comprehensive survey of the molecular mechanism of melatonin on banana seedlings under salt stress conditions, we constructed nine cDNA libraries using the Illumina sequencing platform and generated nine sub transcriptomes. Therefore, RNA sequence analysis was performed on RNA samples from banana seedlings of three treatments (0 mmol/l NaCl, 60 mM of NaCl, and 60 mM of NaCl plus 100 μM melatonin) to assess the transcriptome sequences among three different treatment samples of banana seedlings, and to explore the variation of DEGs and biological metabolic pathways related to melatonin alleviate salt stress response. The Illumina sequencing identified 3,378 DEGs in S and CK, 4,969 DEGs identified in MS and CK, and 1,697 DEGs identified in MS vs. S. There were more differentially expressed genes in S and CK than in MS and CK, indicating that melatonin may induce more stress-related genes in salt stress situation (Figure 3). GO enrichment analysis showed that the melatonin-induced salt resistance involved in the process of biological processes, molecular functions, and cellular components of banana seedlings (Figure 4). Through GO analysis, we classified the functions of the transcripts in different treatments, with a threshold of *p* < 0.01, and divided the three main categories (biological processes, cellular components, and molecular functions) into different subgroups. We also found that DEGs are involved in a variety of metabolic pathways, which are significantly enriched, including amino sugar and nucleotide sugar metabolism, phenylalanine metabolism, cyanoamino acid metabolism, starch, and sucrose metabolism, and linoleic acid metabolism (Supplementary Table 6). Melatonin is widely involved in different metabolic processes in plants, which suggests that these major metabolism and biosynthesis may be involved in the potential mechanism of melatonin of banana seedlings under salt stress. Many significant pathways can help plants against the abiotic and biological stresses reported in previous studies (Goyal et al., 2016; Zhang et al., 2017). We have identified the top ten significant enrichment pathways among the three different treatments through pathway enrichment analysis (*p* < 0.01) (Supplementary Table 5). We also found two pathways, including Phenylpropanoid biosynthesis (ko00940) and Flavonoid biosynthesis (ko00941) were simultaneously and significantly enriched in all three treatments. It is well-known that the Phenylpropane biosynthesis pathway can provide anthocyanins for pigmentation and flavonoids to prevent stress damage (Ferrer et al., 2008).

According to KEGG enrichment analysis results, we make further efforts to analyze the DEGs related to the plant hormone signal transduction pathway (ko04075) in MS and S, including auxin, cytokinin, and abscisic acid in banana seedlings (Figure 5). In our study, the function of ARF as a transcriptional activator of auxin-regulated genes (Ma06_g09030; Ma06_g30810) was upregulated between MS and S (Mano and Nemoto, 2012). The cytokinin signaling mechanism consists of the following part: AHKs (Histidine kinases), AHPs (histidine-containing phosphotransfer protein), and ARRs (response regulators) in the higher plant (Suzuki et al., 2000; Veerabagu et al., 2012). Between MS vs. S, the expression of two genes homologous to A-ARR (Ma06_g19860 Ma08_g29720) in banana seedlings was downregulated, and homologous to AHP (Ma10_g14170, Ma11_g10050) was upregulated, which may be conducive to the growth of banana seedlings. It was revealed that the expression of gibberellin receptor GID1 (Ma06_g17630) was upregulated between MS vs. S, which undoubtedly promoted banana seedling growth. In our study, α-linolenic acid metabolism (Ko00592) and plant hormone signal transduction (Ko04075) were significantly enriched between MS and S, which is the same as that of Kiwifruit (Tan et al., 2019).

Transcription factors (TFs) participate in the response induced by plant stress tolerance and act as upstream regulators to regulate gene expression in the process of plant metabolic pathways (Shan et al., 2013). According to different DNA binding domains, transcription factors in plants can be divided into several families. The transcription factors related to stress resistance mainly include AP2/ERF, NAC, bZIP, MYB, WRKY, and so on. These TFs respond to stress in varying degrees and play an important function in the process of the plant facing with biotic or abiotic stress. Here, we clarified some TFs family members’ genes consist of MYB, NAC, bHLH, and WRKY, and so on in the three treatments. We also compared the expression profiles of TFs between different processes to evaluate the complex signal pathway network (Supplementary Table 7; Figure 6). Transgenic plants overexpressing these TFs can enhance the stress resistance of these transgenic plants (Hao et al., 2011; Shen et al., 2012; Sakuraba et al., 2015; Wang et al., 2018). Some TFs were confirmed to work in melatonin-mediated stress response tolerance. ZAT6 participated in melatonin-mediated antifreeze stress by activating the CBF pathway (Shi and Chan, 2014). MeWRKY79 and MeHsf20 upregulate melatonin biosynthesis by binding to the MeASMT2 promoter, thus endowing cassava with resistance to cassava bacterial blight (*Manihot esculenta*) (Wei et al., 2017). We also found that there were significant differences in TFs expression among the three treatments. The number of upregulated was reduced in treated with NaCl and melatonin in banana seedlings. These TFs might contribute to melatonin alleviating salt stress tolerance of banana seedlings. Overall, melatonin can alleviate the development of banana seedlings through plant hormone regulation (Figure 6). Our results indicated that melatonin could be used in banana production in saline soil.

## Conclusion

In conclusion, the growth results imply that 60 mM NaCl salt stress inhibited the growth of banana seedlings, while exogenous application of 100 μM melatonin could alleviate the symptoms of salt stress and significantly improve the growth of banana seedlings. Physiological data analysis showed that exogenous melatonin could improve the salt stress resistance of banana seedlings by increasing proline content and soluble protein content, slowing down chlorophyll degradation, reducing membrane permeability, restoring relative water content, increasing MDA accumulation, and improving antioxidant enzyme activity. Transcriptome sequencing showed that melatonin-induced salt tolerance of banana seedlings involved in a variety of metabolic pathways, including amino sugar and nucleotide sugar metabolism, phenylalanine metabolism, cyanoamino acid metabolism, starch and sucrose metabolism, and linoleic acid metabolism. Furthermore, some members of the transcription factor family, such as MYB, NAC, bHLH, and WRKY, might contribute to melatonin alleviating salt stress tolerance of banana seedlings. The result laid a basis for further elucidating the mechanism of exogenous melatonin-mediated salt stress resistance in banana seedlings and provides a theoretical basis for using melatonin to improve the salt tolerance of banana seedlings in the future.

## Data availability statement

The data presented in the study are deposited in the NCBI repository, accession number PRJNA846084.

## Author contributions

JW and DL designed the research and wrote and revised the manuscript. JW and YL performed and analyzed most of the experiments. JL, GL, and SW helped to arrange the experimental data. All authors read and approved the final manuscript.
